# Saliva diagnostic utility in patients with type 2 diabetes: Future standard method

**DOI:** 10.2478/jomb-2019-0019

**Published:** 2020-01-23

**Authors:** Marwa Mrag, Asma Kassab, Asma Omezzine, Chebil Raoua Belkacem, Fredj Ismail Fatma Ben, Nabiha Douki, Kechrid Chedia Laouani, Ali Bouslema, Amor Faten Ben

**Affiliations:** 1 University of Monastir, Faculty of Dental Medicine, Oral Health and Oro-Facial Rehabilitation Research Laboratory, Monastir, Tunisia; 2 University of Monastir, Faculty of Pharmacy, Monastir, Tunisia; 3 Sahloul Hospital, Internal Medicine Department, Sousse, Tunisia

**Keywords:** saliva, glucose, urea, type 2 diabetes, dijabetes tipa 2, urea, glukoza, pljuvačka

## Abstract

**Background:**

The purpose of the present study was to assess saliva reliability in diagnosis and monitoring type 2 diabetes instead of blood.

**Methods:**

Blood and unstimulated whole saliva were collected from 300 type 2 diabetic subjects and 300 healthy controls in fasting. Then, the salivary flow rate was calculated. All parameters including glucose, urea, amylase, total protein, albumin, C-reactive protein (CRP), immunoglobulin A (IgA), potassium, calcium and chloride were assessed in the supernatant, using an autoanalyzer. Oral exam was conducted by a single examiner on full mouth excluding third molars. Statistical analysis was performed by the SPSS 20.0 version.

**Results:**

Saliva screening showed that glucose, urea, amylase, total protein, potassium, calcium and chloride were significantly higher in patients compared to controls (p < 0.05). Whereas, the IgA level and salivary flow rate were significantly reduced in patients (p < 0.05). No significant difference was found in albumin and CRP levels (p > 0.05). There was a significant positive correlation between salivary and plasma glucose levels (r = 0.887, and r = 0.900, p < 0.001), as well as, salivary and blood urea (r = 0.586, and r = 0.688, p < 0.001) in patients and controls, respectively.

**Conclusions:**

From this study, saliva could be suggested as a useful diagnostic tool for type 2 diabetes.

## Abstract (sr)

**Uvod:**

Svrha ove studije bila je procena pouzdanosti pljuvačke u dijagnostikovanju i praćenju dijabetesa tip 2 umesto krvi.

**Metode:**

Od 300 osoba sa dijabetesom tip 2 uzeti su krv i nestimulisana ukupna pljuvačka, kao i od 300 zdravih osoba koje su predstavljale kontrolnu grupu i koje su se suzdržale od uzimanja hrane. Zatim je izračunata brzina protoka pljuvačke. Svi parametri, uključujući glukozu, ureu, amilazu, ukupni protein, albumin, C-reaktivni protein (CRP), imunoglobulin A (IgA), kalijum, kalcijum i hlorid su ispitani u supernatantu uz korišćenje autoanalizatora. Oralni pregled je obavila jedna osoba na kompletnu vilicu bez umnjaka. Statistička analiza je urađena kroz program SPSS u verziji 20.0.

**Rezultati:**

Skrining pluvačke je pokazao da su glukoza, urea, amilaza, ukupni protein, kalijum, kalcijum i hlorid bili značajno viši kod pacijenata u poređenju sa kontrolom grupom (p < 0,05). S druge strane, nivo IgA i brzina protoka pljuvačke su bili značajno smanjeni kod pacijenata (p < 0,05). Nije utvrđena značajna razlika u nivoima albumina i CRP (p > 0,05). Utvrđena je značajna pozitivna korelacija između nivoa pljuvačke i glukoze u plazmi (r = 0,887; i r = 0,900, p < 0,001), kao i pljuvačke i uree krvi (r = 0,586, i r = 0,688, p < 0,001) i kod pacijenata i kod kontrolne grupe.

**Zaključak:**

Iz ove studije proističe da se pljuvačka se može predložiti kao korisno dijagnostičko sredstvo za dijabetes tip 2.

## Introduction

Type 2 diabetes, is the most common form of diabetes. In the Tunisian population, it accounts for about 15.1% cases, thereby representing a major public health problem [1].

Type 2 diabetes is a chronic resistance to insulin action in target cells and a further relative insulin deficiency. It results in hyperglycemia which may lead to vascular complications. This chronic metabolic disease mainly affects the heart, kidneys, eyes, and nerves [2] and oral cavity [3]. It has also been associated with salivary gland function impairment triggering consequently oral health homeostasis alteration and generating oral diseases [4]. Diabetic subjects are prone to caries, gingivitis and xerostomia [5].

Diabetes has always been regarded as a challenge to health professionals since it always requires monitoring. Blood analysis is considered as the only conventional method to assess biologic check-up. Nevertheless, the blood draw is invasive, leading to psychological stress for some patients.

Nowadays, researches are focused around setting up noninvasive techniques. Importantly, saliva offers more advantages to be inexpensive; easy to collect, to transport, to store [6], and non-invasive as it has been said by Mandel: »It lacks the drama of blood« [7]. Saliva is an oral heterogeneous biofluid, composed of a variety of constituents that play a crucial role in oral health homeostasis. Furthermore, studies attest that salivary composition and function are affected by both local and systemic changes. In fact, saliva has been well recognized as a mirror of the body health [6]. Thereby, salivary molecules could be strong indicators for predicting, monitoring and diagnosing systemic and local disorders [8] [9]. In this con text, the aim of the present study is to provide a great concern to show saliva diagnostic evidence. Therefore, several biochemical parameters as glucose, urea, amylase, total protein, albumin, electro lytes, Creactive protein (CRP), and immunoglobulin A (IgA) in diabetic subjects are investigated.

## Material and Methods

### Study population

We performed a case-control study of 300 type 2 diabetic patients recruited from the Internal Medicine department, age-sex matched to 300 healthy volunteer subjects recruited from Sahloul Hospital, Sousse, Tunisia. The study protocol was approved by the local Ethics Committee of Sahloul University Hospital conforming to Helsinki Declaration.

All subjects read and signed a written informed consent before their enrollment into the study.

Clinical data was collected via hospital medical files and a semi-structured interview with subjects.

We excluded from the study fully edentulous subjects, smokers, alcoholics, subjects with xerostomia, and people diagnosed with an illness which may affect oral health status.

### Oral examination

Clinical examinations were conducted in Dental Medicine Department of Sahloul University Hospital. Full-mouth assessments on six sites per tooth (mesiobuccal, medio-buccal, disto-buccal, disto-lingual, medio-lingual and mesio-lingual) excluding third molars were performed by a single examiner. Clinical attachment level (CAL) measurements using a standard William's graduated periodontal probe, allowed classification of the periodontitis into mild (1-2 mm CAL), moderate (3-4 mm CAL) or severe (> 5 mm CAL).

The caries status of subjects was evaluated using decayed, missing and filled permanent teeth index (DMFT). Oral hygiene status, tasteimpairment, oral candidiasis, gingivitis, plaque and dental calculus presence were also determined. Xerostomia was evaluated via the Fox test [10].

### Saliva and blood sampling

For each subject, specimens were collected after 8 hours of fasting. Unstimulated whole saliva was collected by the spitting method [11], after rinsing the mouth with water to remove any debris, subjects were asked to spit saliva into a preweighed tube during a 5 min period. The flow rate was calculated and expressed in mL/min by the Navazesh & Kumar method [12]. Venous blood was collected in a sodium fluoride tube for the estimation of glucose; in a lithium heparin tube, for the estimation of albumin, CRP, amylase, urea, total protein, calcium, sodium, potassium and chloride, in a serum tube for the estimation of total IgA; and in EDTA tube for HbA1C determination.

### Biochemical analysis

Before biochemical analysis, a centrifugation was performed for the salivary (5000 rpm/10 min /4°C) as well as blood (3000 rpm/5min/4 °C) specimens.

Blood and salivary IgA concentrations were measured by nephelometry method using the IMMAGE Immunochemistry System (Beckman Coulter, USA). Blood and salivary assays on glucose, urea, amylase, electrolytes and CRP carried out by AU680 (Beckman-Coulter, USA) autoanalyzer (measurements range, low concentration detected and precision in serum for these parameters are summarized in Table 1). Glucose, urea and amylase levels were assessed using enzymatic methods. Albumin, total protein and CPR levels were measured by colourimetric bromocresol, biuret test and turbidimetric analysis, respectively.

**Table 1:** Characteristics of Beckman AU methods NP: Not provided

Parameters	Measurement range		Low concentration detected		Precision: Mean Coefficient of Variation (%CV)	
	In serum	In saliva	In serum	In saliva	In serum	In saliva
Glucose (mmol/L)	0.3–43.3	NP	0.04	NP	0.77	NP
Urea (mmol/L)	1.38–39.44	NP	0.38	NP	2.68	NP
Amylase (U/L)	30–1482	NP	1	NP	1.17	NP
Total protein (g/L)	33.24–118.26	NP	0.77	NP	0.72	NP
Albumin (g/L)	11.7–50.85	NP	0.07	NP	1.98	NP
CRP (mg/L)	10–212	NP	1.57	NP	3.27	NP
Potassium (mmol/L)	1.5–7	NP	NP	NP	0.76	NP
Calcium (mmol/L)	1.03–3.83	NP	0.01	NP	0.95	NP
Chloride (mmol/L)	80 –125	NP	NP	NP	0.71	NP

The bovine serum albumin (BSA) (49 g/L) was used as a standard for salivary assays of albumin and total protein. In brief, a total of 200 μL of saliva was mixed with 800 μL of Bromocresol Green Solution, incubated for 30 min at 20-25 °C and the absorbance at 600 nm was recorded for the determination of salivary albumin concentrations (g/L). For total protein levels, 500 μL of saliva sample was added to 2 mL of Gornall reagent, incubated at room temperature for 10 min and then, absorbance at 545 nm was measured.

Salivary chloride, potassium and calcium levels were assessed by potentiometric and colorimetric Arsenazo methods. Before each measurement of amylase and potassium in salivary samples, dilutions were made by 5 to 500.

Glycated haemoglobin (HbA1c) levels were estimated in blood samples by High-performance liquid chromatography (HPLC) Variant II analyzer (BioRad, USA), and HbA1c criterion (≤ 7) was used to determine the level of disease control.

All biochemical assays were carried out in triplicate in the Biochemistry Laboratory of Sahloul Hospital, Sousse, Tunisia.

## Statistical analysis

Statistical tests were performed using the SPSS 20.0 version. Parameters were evaluated using twoway analysis of variance, then compared by Student's t-test. If their distribution was Gaussian, they were represented as mean ± standard deviation. Otherwise, they were reported as median [min-max] and compared by non-parametric U test of Mann-Whitney. Categorical variables were analyzed by the Pearsonchi square test. Correlation between blood and salivary parameters levels was assessed by Pearson's and Spearman correlation coefficients. Regression equations were used to calculate blood levels from the salivary value. The ROC (Receiver operating characteristic) analysis was used to assess the diagnostic reliability (sensitivity and specificity) of salivary glucose and urea.

## Results

The study included 300 type 2 diabetics and 300 controls with a mean age of 62.05 ± 11.3 and 60.95 ± 8.77 years, respectively. The collected clinical data of the study population are summarized in Table 2.

**Table 2:** Clinical data of diabetics and controls SD: standard deviation, BMI: body mass index, med: median, min: minimum, max: maximum

Parameters	Diabetics (n = 300)	Controls (n = 300)
Sex ratio	0.90	0.72
Age (mean ± SD) (years)	62.05 ± 11.3	60.95 ± 8.77
BMI (mean ± SD) (kg.m^-2^)	28.69 ± 4.68	25.38 ± 1.9
HbA1c (mean ± SD) (%)	8.68 ± 2.36	4.93 ± 0.58
Diabetes duration (mean ± SD) (years)	12.61 ± 5.36	–
HbA1c n (%) ≤ 7: well controlled > 7: poorly controlled	125 (41.67) 175 (58.33)	–
Diabetes treatment n (%) Only on oral hypoglycemic drugs Only on insulin On both	156 (52) 81 (27) 43 (14.3)	–
Family history of diabetes n (%)	240 (72.9)	89 (27.1)
Diabetic complications n (%) retinopathy nephropathy cardiovascular diseases	80 (26.7) 81 (27) 23 (7.7)	–
Elevated blood pressure n (%)	174 (58)	–
Hypercholesterolemia n (%)	108 (36)	–

Oral health status and salivary parameters of the study population are presented in Table 3. Compared to controls, type 2 diabetics exhibited poorer oral hygiene and significantly higher oral complications incidence including xerostomia, halitosis and taste impairment (p < 0.05). A significantly higher score of DMFT index was perceived in diabetics than in controls (p < 0.05). The salivary flow rate was found to be significantly reduced in diabetics compared to controls (p < 0.05).

**Table 3:** Oral health status and salivary parameters ofdiabetics and controls med: median, min: minimum, max: maximum,+: mild, ++: moderate, +++: severe, DMFT: decayed, missing, filled permanent teeth, SD: standard deviation

Parameters	Diabetics (n = 300)	Controls (n = 300)	p value
Number of tooth brushing per day n (%)			0.341
[1–2]	205 (68.3)	194 (64.7)	
[3–4]	95 (31.7)	106 (35.3)	
Xerostomia n (%)	174(58)	96 (32)	< 0.001
Halitosis n (%)	132 (44)	90 (30)	< 0.001
Taste impairment n (%)	148 (49.3)	111 (37)	0.002
Oral Candidiasis n (%)	71 (23.7)	53 (17.7)	0.070
Plaque n (%)	219 (73)	87 (29)	< 0.001
Dental calculus n (%)	224 (74.7)	76 (25.3)	< 0.001
Periodontitis n (%)			< 0.001
(+)	50 (16.8)	0 (0)	
(++)	88 (29.2)	0 (0)	
(+++)	162 (54)	0 (0)	
DMFT index (med [min; max])	10 [1–24]	3 [0–9]	< 0.001
Salivary flow rate (mean± SD)	0.50± 0.13	0.75±0.08	< 0.001

Salivary and blood biochemical parameters in diabetics and controls are given in Table 4.

**Table 4:** Salivary and blood profile in diabetics and controls med: median, min: minimum, max: maximum, SD: standard deviation, **: Correlation is significant at the 0.01 level

Parameters	Saliva			Blood			Blood-Saliva correlation			
	Diabetics (n = 300)	Controls (n = 300)	p	Diabetics (n = 300)	Controls (n = 300)	p	Diabetics (n = 300)		Controls (n = 300)	
							p	r	p	r
Glucose med [min; max] (mmol/L)	0.4 [0.2–5.4]	0.2 [0–0.3]	< 0.001	10 [7–14.1]	4.35 [3.1–5.8]	< 0.001	< 0.001	0.887**	< 0.001	0.900**
Urea med [min; max] (mmol/L)	6.8 [3–27.7]	1.54 [0.88–2.4]	< 0.001	5.85 [2.3–26.6]	4 [2.5–7]	< 0.001	< 0.001	0.586**	< 0.001	0.688**
Amylase med [min; max] (U/L)	143461.5 [136–57907]	1638 [379–2294]	< 0.001	79 [35–136]	77 [17–133]	0.419	0.079	0.102	0. 671	0.017
Total protein med [min; max] (g/L)	0.48 [0.16–2.2]	0.195 [0.10–0.34]	< 0.001	72 [48–85]	71 [55–84]	0.308	0.058	0.109	0.547	–0.035
Albumin med [min; max] (g/L)	0.61 [0.10–2.6]	0.8 [0.4–1.2]	0.881	43 [34–59]	44 [31– 49]	0.068	0.445	–0.044	0.476	–0.041
CRP (mean ± SD) (mg/ L)	4.22±0.57	4.15±0.53	0.140	6.05±0.81	5.94±0.77	0.100	0.070	0.105	0.085	–0.100
Total IgA med [min; max] (g/L)	0.21 [0.07–2.6]	0.57 [0.1–3]	< 0.001	3 [0.8–6.14]	3 [0.8–5.5]	0.376	0.061	0.108	0.205	–0.073
Potassium (mean ± SD) (mmol/L)	28.07±14.02	15.32±3.28	< 0.001	4.24±0.58	4.16±0.51	0.059	0.533	–0.036	0.107	0.093
Calcium (mean ± SD) (mmol/L)	1.52±0.68	0.99±0.27	< 0.001	2.33±0.44	2.39±0.37	0.065	0.199	–0.074	0.232	0.069
Chloride med [min; max] (mmol/L)	13 [8–76]	13 [11–16]	< 0.001	105 [93–115]	105 [99–111]	0.112	0.244	0.067	0.789	0.016

Saliva screening showed a significant increase of the majority of parameters concentrations such as glucose, urea, amylase, total protein and electrolytes including potassium, calcium and chloride in diabetics compared to controls (p < 0.05). However, total IgA concentration was found to be significantly lower in diabetics compared to controls (p < 0.05).

Analysis of the blood profile showed a significantincrease only in glucose and urea in diabetics compared to controls (p < 0.05).

Interestingly, our analysis showed the presence of two significant correlations. The first one was between salivary and blood glucose in diabetics (r = 0.887, p < 0.001) and in controls (r = 0.900, p < 0.001). The second was between salivary and blood urea in diabetics (r =0.586, p < 0.001) and in controls (r = 0.688, p < 0.001).

No correlations were found either in people with diabetes or in controls between salivary and blood albumin, CRP, amylase, total protein, calcium, potassium, chloride and total IgA.

**Figure 1:**
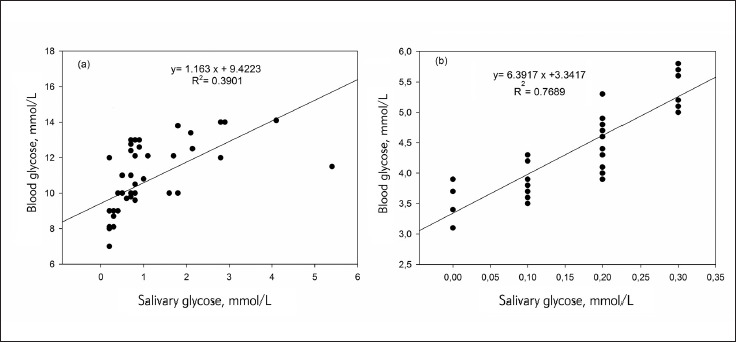
Linear regression analysis correlation between blood glucose and salivary glucose in diabetics (A) and controls (B)

For further analysis concerning the correlation between blood and salivary parameters, particularly glucose and urea, we performed a linear regression correlation in diabetics and controls (Figure 1).

**Figure 2:**
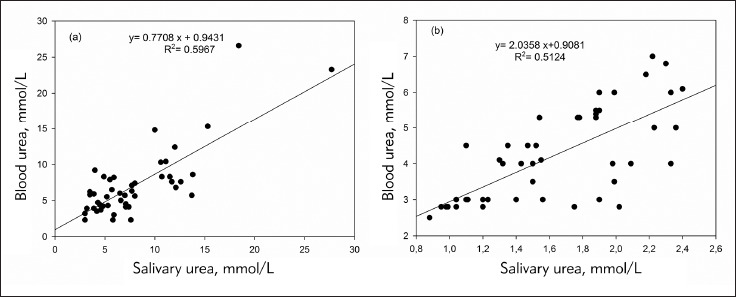
Linear regression analysis correlation between blood urea and salivary urea in people with diabetes (A) and controls (B)

**Figure 3:**
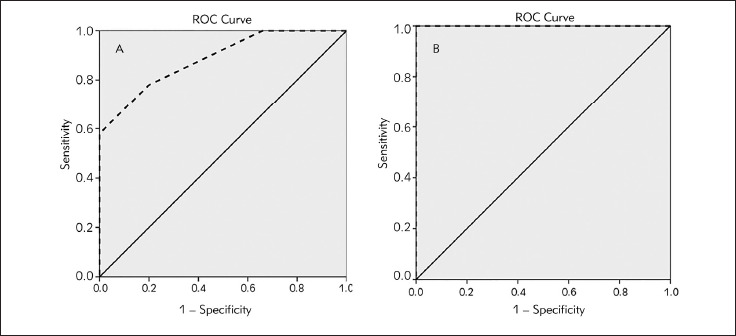
ROC Curve for salivary glucose (A) and salivary urea (B) ROC curve: receiver operating characteristic curve diagonal segments are produced by ties––––– : reference line - - - - - : salivary parameter

The R squared (R2) values were 0.3901 and 0.7689 for diabetics and for controls respectively. In addition, a regression equation was ascertained as y = a (×) + b, for diabetics y (blood glucose) = 1.163 × (salivary glucose) + 9.4223 (Figure 1A). For controls the equation was as y (blood glucose) = 6.3917 × (salivary glucose) + 3.3417, (Figure 1B). Indeed, (Figure 2) showed linear regression correlation between salivary and blood urea in diabetics (Figure 2A) and controls (Figure 2B). The R squared (R2) values were 0.5967 and 0.5124 in diabetics and controls, respectively. For diabetics y (blood urea) = 0.7708 × (salivary urea) + 0.9431 (Figure 2A), and for controls (blood urea) = 2.0358 × (salivary urea) + 0.9081 (Figure 2B). The diagnostic potential of saliva was determined by a ROC analysis (Figure 3A). A cut-off value of salivary glucose was found to be 0.25 mmol/L with a specificity of 80% and a sensitivity of 78% (Figure 3A). Area under the curve (AUC) for salivary glucose was 0.885 with a 95% confidence interval (95% CI) of 0.860-0.911 (p < 0.001) and a standard error of 0.013. For the salivary urea, the cut-off value was found to be 2.7 mmol/L with specificity and sensitivity of 100% (Figure 3B). The AUC for salivary urea was 1.0, with 95% CI of 1.0-1.0 (p < 0.001) and a standard error of 0.000.

## Discussion

Type 2 diabetes is a chronic metabolic disease which often requires continuous monitoring using a stressful conventional method. Therefore, the purpose of the present work was to investigate saliva diagnostic evidence in monitoring type 2 diabetes. Currently, most of type 2 diabetes subjects exhibited several oral complications that hamper the quality of life like xerostomia, halitosis, taste impairment, in addition to a high average of periodontal diseases as well as dental caries. This observation was in accordance with several studies [6] [13] [14] [15] which mentioned a high frequency of oral manifestations related to diabetes due to poor metabolic control. Hence, impairment of salivary gland function and composition was reported to be associated with a reduced salivary secretion which develops xerostomia and increases dental caries susceptibility as well as other oral diseases such periodontal diseases [16].

Despite the received treatments, the metabolic status in our patients remained unbalanced (HbA1c > 7%), and that could explain the elevated blood glucose levels registered in our diabetic subjects. Indeed, persistent hyperglycemia is a major cause of a progressive renal dysfunction [17] which was manifested by the uremia noticed in our study. The hyperglycemia-uremia synergy was in accordance with the reported literature [17] [18].

The elevated salivary glucose and urea levels in diabetics were in accordance with the findings of Lima-Aragao et al. [6]. This could be due to the fact that persistent hyperglycemia alters both blood vessels and basement membrane permeability of salivary gland [19], leading to an increase of glucose percolation as well as other small molecules like urea, which are not secreted by salivary gland [20] from blood to saliva through gingival crevices [21]. The association of salivary glucose increase and the salivary flow rate decrease was reported to be implicated in xerostomia for diabetic patients [22].

The synergistic enhancement of salivary total protein and amylase levels in people with diabetes was in agreement with the study of Abd-Elraheem et al. [23]. Whereas, Indira et al. [24] reported a synergistic reduction of total protein and amylase levels.

Being the first line of defence against pathogens, salivary IgA plays a protective role in diabetic patients with periodontitis [25]. This confirms the significant reduction of total salivary IgA in people with diabetes registered in our study. Such reduction may be due to local immune humoral disruption affected by diabetes and aggravated by periodontitis [25]. This finding was in line with some investigations [25] [26], and contrast with others [6] [23]. Our results showed a significant increase in the all salivary electrolytes levels such as calcium, chloride and potassium in people with diabetes. Electrolytes play a role in dental health, particularly, calcium in enamel remineralization [27].

Similar results concerning calcium [6] [27], chloride and potassium [26] levels were observed. High potassium levels were explained by a modified transport of the electrolyte in the salivary gland caused by dysfunction of Na^+^ -K^+^-ATPase activity in diabetic patients [28].

In our study, saliva screening showed an altered salivary composition in people with diabetes comparing to controls. In fact, it was previously suggested that this alteration would be due to a compromised salivary gland function caused either by autonomic neuropathy, or basement membrane harm, particularly, in poorly controlled diabetes [29] [30].

Importantly, in the present study, the statistical analysis showed a significant positive correlation between salivary and blood glucose in patients (r = 0.887, p < 0.001) as well as in controls (r = 0.900, p < 0.001). Similar results were found when assessing fasting glucose in type 2 diabetic subjects [31] [32] [33]. Moreover, this correlation was also reported in controlled as well as in uncontrolled type 2 diabetics apart from the healthy controls [34] [35]. Whereas, in the study of Archana et al. [22], the correlation between salivary and blood glucose was found only in uncontrolled type 2 diabetic patients. In the study of Abd-Elraheem et al. [23], a significant positive correlation between postprandial blood glucose and salivary glucose was found only in type 2 diabetic subjects. Also, a positive correlation was reported in type 1 diabetic patients [36].

Nevertheless, few studies found a correlation between salivary and blood glucose [6] [37] [38]. Currently, salivary urea was also found to be positively correlated to the blood urea in both diabetics and controls (r = 0.586, p < 0.001 and r = 0.688, p < 0.001) respectively. A similar correlation was described in an earlier study conducted by Sein and Arumaina yagam [39] on patients with either hypertension, diabetes, or chronic renal failure, and in patients undergoing hemo dialysis. Otherwise, the positive correlation between salivary and blood urea was recently reported in chronic kidney disease patients [40].

Linear regression analysis displayed a linear relationship between salivary and blood glucose con centrations. Thus, the established regression equation as y=a(×)+b, allowed to predict blood glucose level from a known salivary glucose value and vice versa. In patients, the equation was blood glucose =1.163 × (salivary glucose) + 9.4223. In controls, the equation was blood glucose=6.3917 × (salivary glucose) + 3.3417.

Therefore, it was noticed that when a salivary level is equal to or greater than 0.25 mmol/L, the subject could be considered as a diabetic with a specificity of 80% and a sensitivity of 78%.

Similarly, a regression formula was performed to predict blood urea from salivary urea and vice versa in both patients and controls. Thus, beyond a salivary urea cut-off value of 2.7 mmol/L with specificity and sensitivity of 100%, the subject could be considered as a diabetic. To the best of our knowledge, no cutoff of salivary urea in type 2 diabetic subjects has been reported in the literature. Salivary urea ascertainment offers an advantage to caried out at any time of the day since it is a stable molecule [41]. Importantly, salivary urea assessment could avoid stressful blood sampling. Besides, it is considered as a nephropathy predictor by revealing the glomerular filtration rate.

The present study showed an area under curve close to 1 with good specificity and sensitivity, suggesting the salivary glucose and urea as a reliable diagnostic test. The performed ROC analysis allowed to validate saliva effectiveness in diabetes diagnosis. Nevertheless, it would be required to check the diagnostic value of the salivary test with the conventional method before adopting it as an alternative diagnostic method [41].

In a previous study, a salivary-blood albumin correlation was observed in both control and diabetic subjects [27]. The same investigation also showed a salivary-blood amylase correlation and salivary-blood total protein correlation only in diabetics [27]. Besides, the authors reported a salivary-blood calcium correlation only in controls [27].

In conclusion, the salivary profile in type 2 diabetic patients with periodontal diseases was altered compared to controls. Our results showed that saliva is of clinical importance as a reliable non-invasive tool for both early diagnosis, and type 2 diabetes mellitus monitoring since both salivary glucose and urea were found to be directly related to their concentration in blood. As such, saliva analysis offers a great and fast tool for dentists to be aware of the systemic patient status for better dental management.

*Acknowledgements*. The authors are grateful to the cooperation of the study participants. They are also grateful to the Sahloul University Hospital Internal Medicine staff for their efforts in sample collection. Many thanks to the Sahloul University Hospital Dental Medicine Department staff for their valuable help.

Authors especially acknowledge the head of Biochemistry Departement and LR12SP11, in Sahloul University Hospital, and their excellent technical team assistance for their significant contribution.

### Conflict of interest statement

The authors state that they have no conflicts of interest regarding the publication of this article.

## List of abbreviations

CRP, C-reactive protein; IgA, immunoglobulinA; CAL, clinical attachment level; DMFT, decayed, missing andfilled permanent teeth; EDTA, ethylenediaminetetraacetic acid;HbA1C, glycated hemoglobin; HPLC, high-performance liquid chromatography; SPSS, Statistical Package for the Social Sciences; ROC, receiver operating characteristic; AUC, area under the curve; CI, confidence interval; SD, standard deviation; BMI, body mass index; Med, median; Min, minimum; Max, maximum; BSA, BovineSerum Albumin; CV, Coefficient of Variation; NP, not provided.
